# (Ultra) High Pressure Homogenization Potential on the Shelf-Life and Functionality of Kiwifruit Juice

**DOI:** 10.3389/fmicb.2019.00246

**Published:** 2019-02-19

**Authors:** Francesca Patrignani, Cinzia Mannozzi, Silvia Tappi, Urszula Tylewicz, Federica Pasini, Vincenzo Castellone, Ylenia Riciputi, Pietro Rocculi, Santina Romani, Maria Fiorenza Caboni, Fausto Gardini, Rosalba Lanciotti, Marco Dalla Rosa

**Affiliations:** ^1^Department of Agricultural and Food Sciences, University of Bologna, Bologna, Italy; ^2^Interdepartmental Centre for Agri-Food Industrial Research, University of Bologna, Bologna, Italy; ^3^Department of Food and Drug, University of Parma, Parma, Italy

**Keywords:** non-thermal treatment, fruit juice, storage, color, antioxidant activity, viscosity

## Abstract

The increasing competition within the food industry sector makes the requisite of innovation in processes and products essential, leading to focus the interest on the application of new processing technologies including high pressure homogenization (HPH) and ultra high pressure homogenization (UHPH). In this context, the present research aimed at evaluating the effects of two UHPH treatments performed at 200 MPa for 2 and 3 cycles on quality and functionality of organic kiwifruit juice stored at three different temperatures, i.e., 5, 15, and 25°C. The results showed that only the treatment performed at 200 MPa for 3 cycles was able to significantly increase the shelf-life of organic kiwifruit juices when stored at refrigeration temperature, avoiding also phase separation that occurred in the sample treated at 0.1 MPa (control) after 20 days of refrigerated storage. The obtained data showed also that the highest applied pressure was able to increase some quality parameters of the juice such as viscosity and luminosity (L^∗^) and increased the availability of total phenol content consequently enhancing the juice total antioxidant activity. The application of a treatment at 200 MPa for 3 cycles allowed to obtain a stable kiwifruit juice for more than 40 days under refrigerated storage. A challenge to implement this technology in food process as full alternative to thermal treatment could be represented by the adoption of pressure level up to 400 MPa followed by the packaging in aseptic conditions.

## Introduction

Mild non-thermal processes have recently drawn considerable attention in the food processing sector, on the account of their ability to combine microbial inhibition with high retention of qualitative, nutritional, and sensory features of raw materials and ingredients. This great interest from industry is generated in response to consumer’s demand for new products characterized by high functionality and sustainability properties. Among the emerging non-thermal processes, such as high pressure processing, pulsed electric field (PEF), high pressure homogenization (HPH) is considered by an extensive literature as one of the most promising alternative to traditional heat treatments for food stabilization ad differentiation of several products such as fermented milks, emulsions, egg-based liquid preparation, and fruit juices (Lanciotti et al., 1994; Wuytack et al., 2002; Diels and Michiels, 2006; Bevilacqua et al., 2009; Patrignani et al., 2009a, 2010, 2013; Zhao et al., 2014; Ferragut et al., 2015).

Mechanisms of microbial inactivation due to HPH processing are the result of different events such as cavitation, shear stress, turbulence, and impingement which arise during the food treatment (Zamora and Guamis, 2015; Patrignani and Lanciotti, 2016). HPH has also been proved to inactivate or modulate the activity of enzymes that cause phase separation in fruit or vegetable juices, to preserve the initial juice color, flavor, and aromas and, finally, to retain the nutritional and functional features of the treated matrices (Patrignani et al., 2013; Błaszczak et al., 2017). Several authors verified the efficacy of this treatment on several matrices such as vegetable milks (Gul et al., 2017), vegetable and fruit juices (Briñez et al., 2006; Betoret et al., 2009; Donsì et al., 2009; Patrignani et al., 2009b; Bevilacqua et al., 2012; Patrignani et al., 2013), milk (Lanciotti et al., 2004a; Hayes et al., 2005), milk-based products (Lanciotti et al., 2004b; Patrignani et al., 2009a; Massoud et al., 2016), and liquid whole egg (Velazquez-Estrada et al., 2008), suggesting also the combination of HPH with further hurdles such as low storage temperature and low pH in order to increase food shelf-life (Briñez et al., 2006; Huang and Kuo, 2015). However, an improvement of the existing HPH technology was also encouraged, resulting in the production of new types of homogenizers and valves, able to reach levels of ultra high pressure homogenization (UHPH) between 200–400 MPa, that allowed to obtain shelf stable products without negative effects on their quality (Zamora and Guamis, 2015).

On the other side, the food industry has highlighted the necessity of tailor-made protocols in order to maximize the shelf-life of HPH and UHPH treated products without detrimental effect on the nutritional functionality. In fact, according to the literature data, HPH and UHPH are reported to reduce nutritional compounds loss (Gul et al., 2017). Some authors evaluated the polyphenols, vitamin C and provitamin A content, and antioxidant activity of apple (Suárez-Jacobo et al., 2012) and orange juices (Velázquez-Estrada et al., 2013) when treated by HPH and UHPH, reporting that this treatment allowed to significantly preserve these compounds compared to the traditional pasteurization process. However, according to our knowledge, scarce references are available in the literature concerning the use of UHPH and its effect on the quality and functionality of organic kiwifruit juice (Yi et al., 2017).

In this framework, the principal aim of this research was to evaluate the effects of two UHPH treatments performed at 200 MPa for 2 and 3 cycles on the overall quality, functionality, and safety of organic kiwifruit juice, immediately after the treatments and during the storage at three different temperatures (5, 15, and 25°C). In order to assess the effects of the proposed treatments, the naturally occurring microbial population, pH, color, viscosity, antioxidant activity, and total phenol content were investigated on the UHPH juice samples and their controls (samples treated at 0.1 MPa). Moreover, the presence of some natural occurring pathogenic species such as *Listeria monocytogenes*, *Salmonella* spp., and *Escherichia coli* was also evaluated during the kiwifruit juice storage.

## Materials and Methods

### Kiwifruit Juice Preparation and (Ultra) High Pressure Homogenization Treatments

Organic kiwifruits (*Actinidia deliciosa* cultivar “Hayward”) were bought on a local market located in Cesena (Italy) and properly stored until the laboratory trials. They were sorted by homogeneous size of 40 mm diameter and a length of 80 mm and refractometric index of 13 ± 1 °Brix. The raw organic kiwifruit juice was obtained by using a lab extractor (Russel Hobbs, 27700-56) and divided in three 5-L batches and subjected, after eliminating the seeds, to different UHPH treatments performed at 0.1 MPa (used as control), 200 MPa for 2 cycles, and 200 MPa for 3 cycles. For all the UHPH treatments, a PANDA high pressure homogenizer (GEA, Parma, Italy), able to reach 220 MPa and provided of a thermal exchanger and a R-type valve was used. The valve assembly comprised a ceramic ball-type impact head, a stainless steel large inner diameter impact ring, and a carbide passage head made of tungsten. The homogenizer was previously washed with 1% NaOH water solution, hot water, and finally refrigerated sterilized water. The inlet temperature of the juice was about 4°C and the increase rate of temperature was about 2°C/10 MPa. The maximum temperature reached during the most severe UHPH treatment was about 44°C, measured by a temperature probe inside the equipment. The controls and treated samples were collected in 250 mL sterilized glass bottles, stored at 5, 15, and 25°C and analyzed over time.

### Microbiological Analyses and pH

The cell loads of naturally occurring yeasts, total coliforms, and lactic acid bacteria were counted by plate counting on Sabouraud Dextrose Agar (Oxoid Ltd., Basingstoke, United Kingdom), Violet Red Bile Agar (Oxoid Ltd.), and de Man, Rogosa, and Sharpe Agar (Oxoid Ltd.), respectively. Decimal dilutions of the samples, performed in Ringer solution [0.9% (w/v) NaCl], were inoculated in Petri dishes and incubated 48 h at 25°C for yeasts, 48 h at 37°C for Lactobacilli, and 24 h at 37°C for total coliforms. Moreover, at each sampling time, the presence of *L. monocytogenes*, *Salmonella enteritidis*, and *E. coli* was assessed in all the juice samples. The presence of the three pathogenic species was investigated according to the ISO methods ISO 6579-1:2017 (2017); ISO 11290-1:2017 (2017); and ISO 16649-3:2015 (2015), respectively.

The pH was measured immediately after juice treatments and during the storage by using a pH-meter Basic 20 (Crison Instruments, Barcelona, Spain).

### Viscosity and Color Analyses

Viscosity of juices was measured by a vibrational viscometer (Viscosilite 700 Hydramotion), previously calibrated with distilled water (viscosity = 1cP).

Color of kiwifruit juice samples was measured using a spectrophotocolorimeter HUNTERLAB ColorFlexTM, mod. A60-1010-615 (Reston, Virginia). For each sample, L^∗^, a^∗^, and b^∗^ parameters from CIELAB scale were measured.

### Determination of Total Phenolic Content (TPC) and Total Antioxidant Capacity (TAC)

Kiwifruit juice samples were analyzed without any extraction using a UV-1601 spectrophotometer from Shimadzu (Duisburg, Germany). Each sample and calibration point were analyzed in three replicates (*n* = 3). The TPC of samples was assessed by means of the Folin-Ciocalteu method (Singleton and Rossi, 1965). The samples absorbances were measured at 750 nm and the phenolic content was calculated on the basis of the gallic acid calibration curve (from 30 to 1000 μg/mL). The results were expressed as mg/100 mL of juice.

To determine the TAC, the ABTS and DPPH assays were performed. The ABTS assay was performed as described by Laporta et al. (2007), while the DPPH assay was evaluated according to Bonoli et al. (2004). The decrease in absorbance was assessed at 517 nm in the 0–30 min range (at 25°C). The values obtained for both TAC assays were compared to the concentration–response curve of the standard Trolox and expressed as μmol of Trolox equivalent (TE)/100 mL.

### Data Analysis

The data are the means of two independent experiments and three repetitions and were analyzed using Statistica software (8.0; StatSoft., Tulsa, OK, United States) by two-way ANOVA followed by Tukey honest significant difference (HSD) test at *p* < 0.05 level to monitor changes over time as well as differences among treatments.

## Results

### Microbial Inactivation and pH

In Table 1, the cell loads of naturally occurring yeasts immediately after the different UHPH treatments and during storage at different temperature are reported. The UHPH treatments adopted were able to reduce the initial level of naturally occurring yeasts (2.4 log CFU/mL) under the detection limit (1 log CFU/mL), immediately after the process. During the storage at 5°C, the control juice reached the microbiological spoiling threshold fixed at 6 log CFU/mL between 27 and 32 days. However, the phase separation in the juice was observed already at 20 days of storage at 5°C.

**Table 1:** Yeast cell loads (log CFU/mL) detected in organic kiwifruit juices immediately after the treatments and during storage at 5, 15, and 25°C in relation to the pressures applied.

Cell load (log CFU/mL)							
**5°C**							

	**T0**	**T5**	**T16**	**T26**	**T33**	**T40**	

0.1 MPa	2.4 ± 0.2	2.5 ± 0.1	3.9 ± 0.2	4.6 ± 0.6^a^	–^∗^	–^∗^	
200 MPa × 2 cycles	^∗∗^	^∗∗^	^∗∗^	1.5 ± 0.1^b^	2.3 ± 0.2	4.0 ± 0.5	
200 MPa × 3 cycles	^∗∗^	^∗∗^	^∗∗^	^∗∗^	^∗∗^	^∗∗^	

**15°C**							

	**T0**	**T2**	**T5**	**T7**	**T9**	**T12**	**T14**

0.1 MPa	2.4 ± 0.2	3.0 ± 0.3	3.2 ± 0.6	4.7 ± 0.4	–^∗^	–^∗^	–^∗^
200 MPa × 2 cycles	^∗∗^	^∗∗^	^∗∗^	^∗∗^	1.5 ± 0.1	–^∗^	–^∗^
200 MPa × 3 cycles	^∗∗^	^∗∗^	^∗∗^	^∗∗^	^∗∗^	4.4 ± 0.4	5.9 ± 0.3

**25°C**							

	**T0**	**T2**	**T5**	**T7**	**T9**	

0.1 MPa	2.4 ± 0.2	4.7 ± 0.2	–^∗^	–^∗^	–^∗^	
200 MPa × 2 cycles	^∗∗^	^∗∗^	–^∗^	–^∗^	–^∗^	
200 MPa × 3 cycles	^∗∗^	^∗∗^	^∗∗^	2.0 ± 0.3	5.4 ± 0.4	


*^∗^Not performed because the juice spoiled.*

*^∗∗^Under the detection limit.*

*Means followed by different letters means significant differences (*p* < 0.05) among samples at each day of storage.*

On the contrary, the naturally occurring yeasts present in the organic kiwifruit juice were not able to recover after the treatment at 200 MPa for 3 cycles at 5°C, while their potential growth was reduced after the treatment at 200 MPa for 2 cycles. In fact, the spoiling threshold was never achieved for this samples and the yeast cell load after 40 days of storage was 4 log CFU/mL.

As expected, as the sample storage temperature increased, a decrease of the juice shelf-life was observed. The samples submitted to the most intense treatment (200 MPa × 3 cycles) spoiled after 14 and 9 days when the storage temperature was 15 and 25°C, respectively. The control sample (treated at 0.1 MPa) spoiled between 7 and 9 days at 15°C and only after 5 days at 25°C.

In the kiwifruit juice treated at 200 MPa for 2 cycles, the yeast cell loads reached the spoilage threshold between 10 and 12 days at 15°C.

For all the considered samples and storage temperatures, total coliforms and lactic acid bacteria never exceeded 1 and 1.5 log CFU/mL, respectively (data not shown). *L. monocytogenes, Salmonella* spp., and *E. coli* were never found in the samples (data not shown).

In Table 2, the pH values of the samples, in relation to the UHPH treatments applied and the storage temperature, are reported. The application of the UHPH treatments in the juice determined a decrease in pH values, which was more pronounced in samples treated with most intense HPH treatment. However, independently on the storage temperature, the sample pH decreased over time.

**Table 2:** pH values detected in organic kiwifruit juices immediately after the treatments and during storage at 5, 15, and 25°C in relation to the pressures applied.

5°C							
	**T0**	**T5**	**T16**	**T26**	**T33**	**T40**

0.1 MPa	3.34 ± 0.01^a^	3.28 ± 0.02^a^	3.14 ± 0.01	3.10 ± 0.03	–^∗^	–^∗^
200 MPa × 2 cycles	3.27 ± 0.01^b^	3.17 ± 0.02^b^	3.12 ± 0.01	3.09 ± 0.01	3.09 ± 0.02	3.17 ± 0.02
200 MPa × 3 cycles	3.25 ± 0.02^b^	3.15 ± 0.02^b^	3.03 ± 0.01	3.06 ± 0.01	3.07 ± 0.01	3.15 ± 0.02

**15°C**							

	**T0**	**T2**	**T5**	**T12**	**T14**

0.1 MPa	3.34 ± 0.01^a^	3.24 ± 0.02^a^	3.19 ± 0.01^a^	–^∗^	–^∗^
200 MPa × 2 cycles	3.27 ± 0.01^b^	3.24 ± 0.01^a^	3.18 ± 0.01^a^	–^∗^	–^∗^
200 MPa × 3 cycles	3.25 ± 0.02^b^	3.22 ± 0.01^a^	3.13 ± 0.01^b^	3.10 ± 0.02	3.05 ± 0.01

**25°C**							

	**T0**	**T2**	**T5**	**T7**	**T9**	
0.1 MPa	3.34 ± 0.01^a^	3.20 ± 0.01^a^	–^∗^	–^∗^		
200 MPa × 2 cycles	3.27 ± 0.01^b^	3.19 ± 0.02^a^	–^∗^	–^∗^		
200 MPa × 3 cycles	3.25 ± 0.02^b^	3.20 ± 0.02^a^	3.18 ± 0.01	3.04 ± 0.02	3.02 ± 0.02	


*^∗^Not performed because the juice spoiled.*

*Means followed by different letters means significant differences (*p* < 0.05) among samples at each day of storage.*

### Viscosity and Color Analyses

In Table 3, the viscosity values recorded for organic kiwifruit juice, in relation to the UHPH treatments applied and storage temperature, are reported. Treatments at 200 MPa × for 2 and 3 cycles resulted in a higher viscosity compared to the control kiwifruit juice. In general, during the storage at 5°C, a decrease of viscosity was observed in all samples, which was more pronounced for control samples and those treated with 200 MPa × 2 cycles. Moreover, while in the control sample the separation of the phases was observed at 20 days, the reduction of the macromolecule size in the treated samples induced a delay in separation and sedimentation. Juices stored at higher temperatures maintained a similar viscosity during the entire period, which was 14 days for samples stored at 15°C and 7 days for those stored at 25°C.

**Table 3:** Viscosity (cP) of organic kiwifruit juices immediately after the treatment and during storage at 5, 15, and 25°C in relation to the pressure applied.

5°C							
	**T0**	**T5**	**T16**	**T26**	**T33**	**T40**

0.1 MPa	1.6 ± 0.1^b^	1.7 ± 0.1^c^	1.4 ± 0.1^b^	1.1 ± 0.0^b^	–^∗^	–^∗^
200 MPa × 2 cycles	2.0 ± 0.1^a^	2.5 ± 0.2^a^	2.3 ± 0.2^a^	1.2 ± 0.1^a^	1.2 ± 0.1^b^	1.3 ± 0.1^b^
200 MPa × 3 cycles	1.8 ± 0.1^ab^	2.2 ± 0.1^b^	2.1 ± 0.1^a^	1.3 ± 0.1^a^	1.5 ± 0.1^a^	1.7 ± 0.2^a^

**15°C**							

	**T0**	**T2**	**T5**	**T12**	**T14**

0.1 MPa	1.6 ± 0.1^b^	1.5 ± 0.2^b^	1.5 ± 0.1^a^	–^∗^	–^∗^
200 MPa × 2 cycles	2.0 ± 0.1^a^	2.2 ± 0.1^a^	1.5 ± 0.1^a^	–^∗^	–^∗^
200 MPa × 3 cycles	1.8 ± 0.1^ab^	2.1 ± 0.1^a^	1.6 ± 0.2^a^	1.8 ± 0.1	1.9 ± 0.1

**25°C**							

	**T0**	**T2**	**T5**	**T7**		

0.1 MPa	1.6 ± 0.1^b^	1.5 ± 0.1^b^	–^∗^	–^∗^		
200 MPa × 2 cycles	2.0 ± 0.1^a^	1.8 ± 0.1^a^	–^∗^	–^∗^		
200 MPa × 3 cycles	1.8 ± 0.1^ab^	1.8 ± 0.1^a^	1.8 ± 0.2	1.8 ± 0.1		


*^∗^Not performed because the juice spoiled.*

*Means followed by different letters means significant differences (*p* < 0.05) among samples at each day of storage.*

Table 4 shows the color parameters measured in control and treated samples during storage at three different temperatures. Lightness (L^∗^) of fresh kiwifruit juice was 33.40. The HPH treatments caused a significant increase of this parameter in comparison to the control samples. Concerning a^∗^ and b^∗^ parameters, respectively, the red/green and the yellow/blue parameter, both samples treated at 200 MPa showed lower values compared to the control sample. During the storage at all considered temperatures, a slight decrease of L^∗^ together with increasing of a^∗^ was observed, while b^∗^ remained almost unchanged in control and 200 MPa × 2cycles treated samples. The samples pressured for 3 cycles presented similar color during the entire storage period.

**Table 4:** Lightness (L^∗^), a^∗^, and b^∗^ values of organic kiwifruit juices immediately after the treatment and during storage at 5, 15, and 25°C in relation to the pressure applied.

L^∗^							
**5°C**							

	**T0**	**T5**	**T16**	**T26**	**T33**	**T40**

0.1 MPa	33.4 ± 0.7^b^	34.6 ± 0.6^b^	30.3 ± 0.4^b^	30.5 ± 0.2^b^	–^∗^	–^∗^
200 MPa × 2 cycles	38.68 ± 0.08^a^	38.6 ± 0.6^a^	36.5 ± 0.2^a^	35.5 ± 0.8^a^	35.4 ± 0.3^a^	35.2 ± 0.4^b^
200 MPa × 3 cycles	38.9 ± 0.2^a^	38.5 ± 0.5^a^	36.2 ± 0.1^a^	36.5 ± 0.8^a^	36.9 ± 0.4^a^	37.7 ± 0.2^a^

**15°C**							

	**T0**	**T2**	**T5**	**T12**	**T14**	

0.1 MPa	33.4 ± 0.7^b^	32.3 ± 0.8^b^	–^∗^	–^∗^	–^∗^	
200 MPa × 2 cycles	38.68 ± 0.08^a^	38.1 ± 0.4^a^	–^∗^	–^∗^	–^∗^	
200 MPa × 3 cycles	38.9 ± 0.2^a^	38.9 ± 0.2^a^	37.99 ± 0.01	36.3 ± 0.2	37.1 ± 0.5	

**25°C**							

	**T0**	**T2**	**T5**	**T7**		

0.1 MPa	33.4 ± 0.7^b^	33.4 ± 0.7^b^	–^∗^	–^∗^		
200 MPa × 2 cycles	38.68 ± 0.08^a^	38.68 ± 0.07^a^	–^∗^	–^∗^		
200 MPa × 3 cycles	38.9 ± 0.2^a^	38.9 ± 0.2^a^	37.5 ± 0.3	36.4 ± 0.2		

**a^∗^**							

**5°C**							

	**T0**	**T5**	**T16**	**T26**	**T33**	**T40**

0.1 MPa	–2.4 ± 0.3^a^	–1.8 ± 0.1^a^	–2.7 ± 0.2^a^	–2.1 ± 0.2^a^	–^∗^	–^∗^
200 MPa × 2 cycles	–3.7 ± 0.2^b^	–4.6 ± 0.2^b^	–3.4 ± 0.1^b^	–2.9 ± 0.1^b^	–2.8 ± 0.2^a^	–2.7 ± 0.1^a^
200 MPa × 3 cycles	–3.4 ± 0.3^b^	–4.4 ± 0.2^b^	–3.7 ± 0.2^b^	–3.2 ± 0.2^c^	–3.2 ± 0.2^b^	–3.15 ± 0.07^b^

**15°C**							

	**T0**	**T2**	**T5**	**T12**	**T14**	

0.1 MPa	–2.4 ± 0.3^a^	–3.4 ± 0.2^a^	–^∗^	–^∗^	–^∗^	
200 MPa × 2 cycles	–3.7 ± 0.2^b^	–4.3 ± 0.2^b^	–^∗^	–^∗^	–^∗^	
200 MPa × 3 cycles	–3.4 ± 0.3^b^	–4.2 ± 0.1^b^	–3.7 ± 0.1	–3.6 ± 0.1	–3.7 ± 0.2	

**25°C**							

	**T0**	**T2**	**T5**	**T7**		

0.1 MPa	–2.4 ± 0.3^a^	–2.4 ± 0.3^a^	–^∗^	–^∗^		
200 MPa × 2 cycles	–3.7 ± 0.2^b^	–3.7 ± 0.1^b^	–^∗^	–^∗^		
200 MPa × 3 cycles	–3.4 ± 0.3^b^	–3.4 ± 0.3^b^	–3.0 ± 0.2	–2.8 ± 0.1		

**b^∗^**							

**5°C**							

	**T0**	**T5**	**T16**	**T26**	**T33**	**T40**

0.1 MPa	16.3 ± 0.6^a^	10.9 ± 1.0^b^	13.9 ± 0.5^a^	15.0 ± 0.3^a^	–^∗^	–^∗^
200 MPa × 2 cycles	14.0 ± 0.5^ab^	13.8 ± 0.7^a^	13.1 ± 0.6^a^	15.1 ± 0.2^a^	15.3 ± 0.3^a^	15.9 ± 0.3^a^
200 MPa × 3 cycles	12.6 ± 0.6^b^	12.6 ± 0.7^a^	11.6 ± 0.2^b^	13.1 ± 0.4^b^	14.7 ± 0.2^b^	15.37 ± 0.04^b^

**15°C**							

	**T0**	**T2**	**T5**	**T12**	**T14**	

0.1 MPa	16.3 ± 0.6^a^	15.1 ± 0.3^a^	–^∗^	–^∗^	–^∗^	
200 MPa × 2 cycles	14.0 ± 0.5^ab^	13.2 ± 0.4^b^	–^∗^	–^∗^	–^∗^	
200 MPa × 3 cycles	12.6 ± 0.6^b^	13.0 ± 0.4^b^	11.1 ± 0.2	11.0 ± 0.2	12.5 ± 0.5	

**25°C**							

	**T0**	**T2**	**T5**	**T7**		

0.1 MPa	16.3 ± 0.6^a^	16.3 ± 0.6^a^	–^∗^	–^∗^		
200 MPa × 2 cycles	14.0 ± 0.5^ab^	14.0 ± 0.3^ab^	–^∗^	–^∗^		
200 MPa × 3 cycles	12.6 ± 0.6^b^	12.6 ± 0.6^b^	12.3 ± 0.2	11.9 ± 0.4		


*^∗^Not performed because the juice spoiled.*

*Means followed by different letters means significant differences (*p* < 0.05) among samples at each day of storage.*

### Total Phenolic Content (TPC) and Total Antioxidant Capacity (TAC)

**FIGURE 1:**
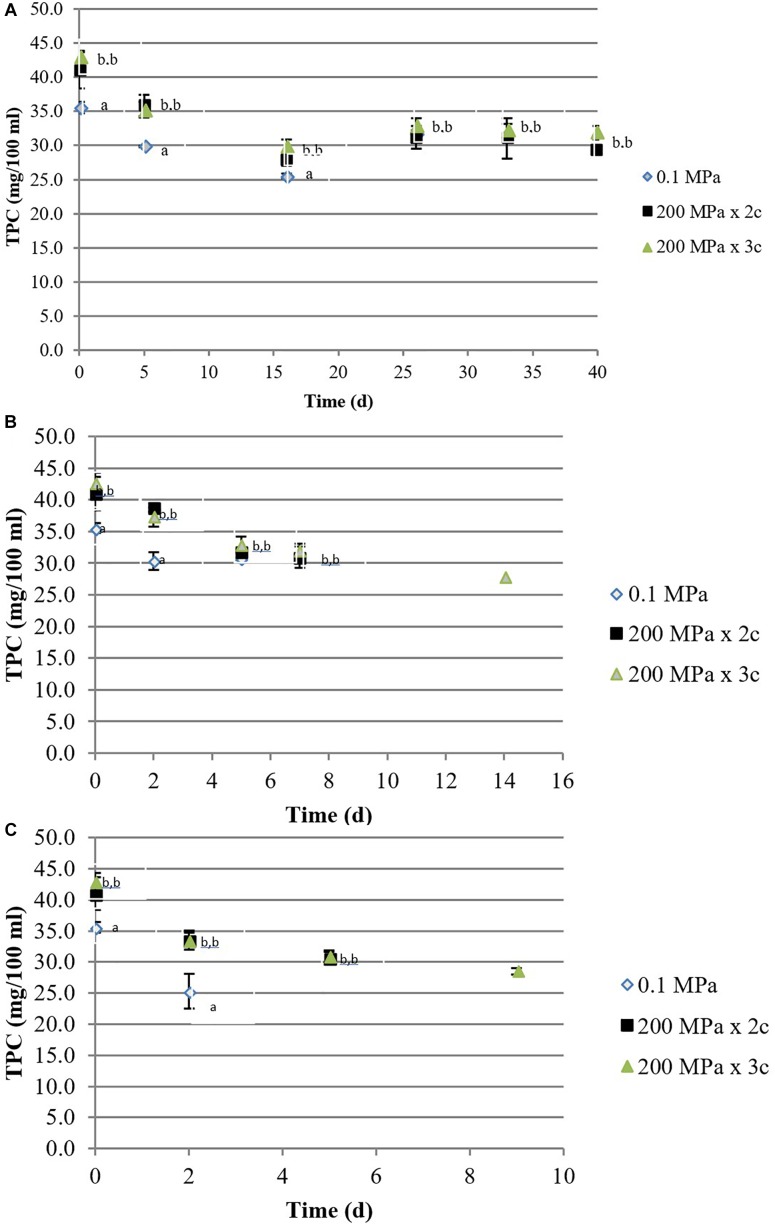
Total phenolic compounds content (mg/100 mL) of organic kiwifruit juices in relation to the high pressure applied, during storage at 5 **(A)**, 15 **(B)**, and 25°C **(C)**. Means followed by different letters means significant differences (*p* < 0.05) among samples at each day of storage.

The total phenolic content of most HPH treated kiwifruit juices significantly increased with respect to the controls from 35 to 42 mg/100mL of juice. During the storage at 5°C, TPC decreased slightly, mainly during the first 15 days, although samples treated at 200 MPa for 3 cycles did not show significant differences (*p* < 0.05; Figure 1A). During the storage at higher temperatures, TPC values decreased in all the samples although the highest values were observed in HPH samples (Figure 1B,C).

**FIGURE 2:**
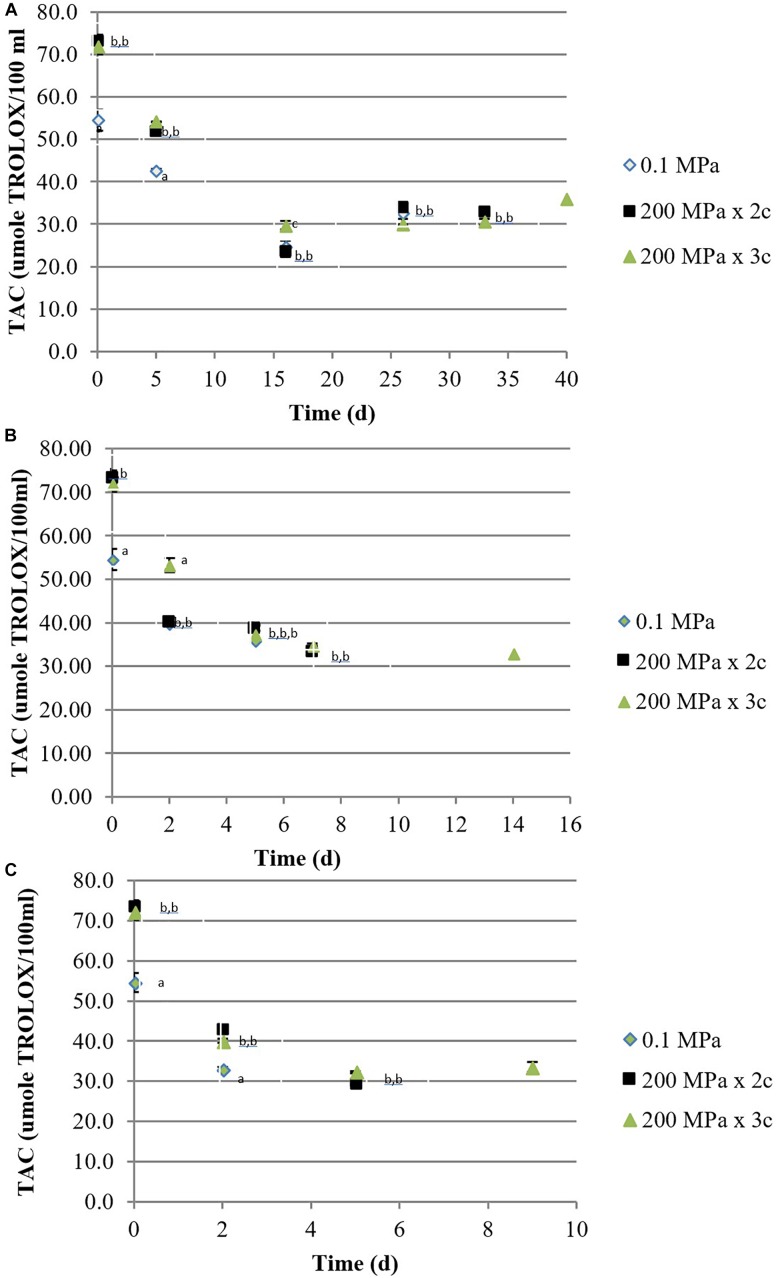
Total antioxidant activity [μmol of Trolox equivalent (TE)/100 mL] by DPPH assay of organic kiwifruit juices in relation to the high pressure applied, during storage at 5 **(A)**, 15 **(B)**, and 25°C **(C)**. Means followed by different letters means significant differences (*p* < 0.05) among samples at each day of storage.

Both treated juices presented a significantly higher antioxidant activity, measured by DPPH, compared to the control sample. As previously observed for TPC, also the antioxidant activity decreased over storage in all the samples, independently on the temperature (Figure 2A–C). ABTS results (data not shown) followed the same trend with an interesting positive Pearson’s correlation with the DPPH method: *r*^2^ = 0.913 *p* < 0.0001, *r*^2^ = 0.923 *p* < 0.0001, and *r*^2^ = 0.983 *p* < 0.0001, for 0.1 MPa, 200 MPa × 2 cycles, and 200 MPa × 3 cycles, respectively.

## Discussion

In the present investigation, the effects of two UHPH treatments performed at 200 MPa for 2 and 3 cycles were investigated on the microbiological stability, color, viscosity, and functionality (availability of polyphenols and antioxidant activity) of organic kiwifruit juice during storage at 5, 15, and 25°C. The performed UHPH treatments promoted an instantaneous reduction of naturally occurring yeasts, reaching values under the detection limit according to the used sampling method. According to the available literature data, the microbial inactivation effect of the HPH processing is influenced by several factors, such as the level of applied pressure and the number of cycles, the different sensitiveness of the microorganisms present and the chemic-physical characteristics of the matrix (Diels and Michiels, 2006; Zamora and Guamis, 2015; Patrignani and Lanciotti, 2016). It is also fundamental to consider the effect of temperature during dynamic pressure treatments, since, an increase of around 2.0°C per 10 MPa can be observed in the food matrix during homogenization. However, Floury et al. (2004) and Pinho et al. (2011) did not observe such temperature increase upon HPH treatment, possibly because of the extremely short time of the treatment duration (less than 1 s). However, in the present research, to minimize the product temperature increase, generated during the treatment, and its effects, a thermal exchanger was applied in order to avoid exceeding the temperature of 44°C.

As microbiological threshold for the kiwifruit juice spoilage, in accordance with the literature data, a yeast cell load level of 6 log CFU/mL was considered. Indeed, yeasts represent the main spoiling agents for this kind of products, characterized by low pH and high sugar content (Donsì et al., 2009; Patrignani et al., 2010, 2013). Although all the UHPH treatments reduced the initial yeast cell loads under the detection limit, it is clear, according to the data, that the applied pressure, especially 200 MPa for 2 cycles, induced sub-lethal damages on the yeast population that was able to recover as function of the storage temperature adopted and level of pressure. Moreover, results suggest that HPH efficiency for microbial inactivation is influenced by various factors, including not only the matrix characteristics and processing parameters, but also the physiological diversity within a microbial population (Ferragut et al., 2015), probably characterized also by different stress resistance and ability to recover. For this reason, in order to validate the effectiveness of a new treatment, the estimation of resistant cells, at the viable but not culturable (VBNC) state, within a microbial population, must be also taken into consideration (Arioli et al., 2019). In the present research, the combination of a UHPH treatment at 200 MPa for 3 cycles and the product refrigeration during storage resulted in a stable and safe organic kiwifruit juice for more than 40 days, without detrimental effects on color, viscosity, and antioxidant activity. The decrease in pH observed in HPH treated kiwifruit juices is in accordance with the data obtained by several authors and can be attributed to the modification of the equilibriums between salts induced by the HPH treatment (Patrignani et al., 2009b, 2013).

From a technological point of view, the increase of viscosity of organic kiwifruit juice in relation to the UHPH treatment applied is a very promising result. Treatments at 200 MPa both for 2 and 3 cycles resulted in a higher viscosity compared to the control kiwifruit juice. This increase was probably due to the structural modification induced by the UHPH treatment, as observed also by Yan et al. (2017) in tomato juice. HPH promotes the disarrangement of the cell clusters into single cells and/or cell fragments. The release and solubilization of cell wall constituents, such as pectin and proteins, cause the increase of the volume fraction of particles and lead to the improvement of particle interactions, thus increasing viscosity (Thakur et al., 1995). However, a decrease in viscosity after HPH treatment has been reported for orange juice (Leite et al., 2014) as well as for banana juice (Calligaris et al., 2012).

Karacam et al. (2015) observed a higher viscosity (gel like structure) in strawberry juice treated at 100 MPa for 2 passes (cycles), compared to 5 passes. According to the authors, the temperature increase during the treatment at 100 MPa × 2 passes reached the optimal temperature for the activation of PME (43°C). Similar results were also observed on mango and apricot juice after HPH depending on pressure increase, inlet temperature, and passes number (Patrignani et al., 2013; Zhou et al., 2017). However, in the present study, the number of passes did not seem to have an influence on the viscosity, probably because temperature increase was similar for both treatments.

Lightness of fresh kiwifruit juice was similar to the value reported by Islam et al. (2012) for organic kiwifruit juice (L^∗^ = 32.00). HPH treatment caused a significant increase of this parameter in comparison to the control samples, which could be due to the higher light scattering properties of smaller size particles (Calligaris et al., 2012). A similar result was also observed by Yi et al. (2017) on apple juice with 50% of kiwifruit addition upon the application of dynamic pressure. Although some authors observed a fair decrease in L^∗^ and an increase in a^∗^ parameter in kiwifruit puree (Fernández-Sestelo et al., 2013) and mango juice (Zhou et al., 2017), in our research, the hyperbaric treatments demonstrated to be able to enhance the typical green color of kiwifruit juice.

The samples pressured with 3 cycles presented similar color during the entire storage period. Similar results were observed by Calligaris et al. (2012) in banana juice stored for 30 days. Lightness of the homogenized banana juice samples decreased only after 20 days of storage; however, homogenized juice remained always lighter than the untreated one during the entire storage period. In our study, the evolution of color in samples stored at the higher temperatures (15 and 25°C) could not be verified due to the juice spoilage already after few days. However, Guan et al. (2016) observed that storage of mango juice at room temperature promotes a greater decrease of lightness and increase of redness compared to storage at 4°C, induced by faster browning reactions. According to the literature data, kiwifruit juice includes a large variety of functional components such as phenolic compounds, antioxidants, potassium, vitamin C, vitamin E, and fibers (Fernández-Sestelo et al., 2013). Moreover, kiwifruit intake is reported to increase cytokine production and exert antioxidant effects (Iwasawa et al., 2010). Unfortunately, processes involving thermal treatments strongly decrease the product’s quality and functionality due to changes induced in thermolabile phytocompounds (Błaszczak et al., 2017). In the present research, the application of UHPH determined a significant increase of the availability of total polyphenols. These data are in agreement with previous literature reports which suggest that the HPH and UHPH process can increase extractability of antioxidant components by breaking down of the cell walls components (Patras et al., 2009a,b). Moreover, an increase in homogenization pressures resulted in a better retention of bioactive compound during storage in low pulp mandarin juice at 20 and 100 MPa (Betoret et al., 2017). Similarly, in the present research, the use of 200 MPa for 3 cycles determined, during the storage, a slower reduction of total polyphenols in kiwifruit juice. Moreover, an increase of the initial antioxidant activity was observed in the samples treated by HPH, independently by the level of pressure applied. These results may be explained by a partial inactivation of polyphenoloxidase and peroxidise enzymes involved in the degradation of phenolic compounds in vegetable matrix (Guan et al., 2016) as observed by Bot et al. (2018) on apple juice treated at 150 MPa for 10 passes.

On the other hand, the antioxidant activity of the treated juice was found to decrease during storage, indicating an incomplete enzyme inactivation (Betoret et al., 2017).

## Conclusion

Since juices stabilized by UHPH are not yet available in the market, the present research presents high industrial relevance, as it provides useful information related to the processing conditions that can allow to obtain safe kiwifruit juices with prolonged shelf-life.

The application of a treatment at 200 MPa for 3 cycles allowed to obtain a stable kiwifruit juice for more than 40 days under refrigerated storage and to extend the shelf-life of 1 week at room temperature with respect to the control, increasing at the same time the polyphenols availability and its antioxidant activity, and allowing to better retain the color.

A further challenge to implement this technology for fluid decontamination as a full alternative to thermal treatment could be represented by the introduction of new generation equipment capable to reach 400 MPa in the traditional process lines also endowed of packaging system in aseptic conditions. Moreover, in the perspective of industrial applications, studies based on predictive microbiology, as well as a deeper comprehension of the action mechanisms of UHPH on the physiological state of microbial cell, play a key role for process optimization.

## Author Contributions

CM, ST, UT, FeP, VC, and YR contributed to the lab analyses. FrP, PR, and SR put in writing the manuscript and performed data analysis. MC, FG, RL, and MDR contributed to the development of research work plan.

## Conflict of Interest Statement

The authors declare that the research was conducted in the absence of any commercial or financial relationships that could be construed as a potential conflict of interest.
